# Why is the Earth System Oscillating at a 6-Year Period?

**DOI:** 10.1007/s10712-024-09874-4

**Published:** 2025-02-10

**Authors:** Anny Cazenave, Julia Pfeffer, Mioara Mandea, Véronique Dehant, Nicolas Gillet

**Affiliations:** 1 Université Toulouse III, LEGOS (CNES/CNRS/IRD/UT3), 18 Avenue Edouard Belin, 31401 Toulouse, Cedex 9, France; 2 Magellium, 1 Rue Ariane, 31520 Ramonville-Saint-Agne, France; 3 Centre National d’Etudes Spatiales, CNES, 2 Place Maurice Quentin, Paris, France; 4 Royal Observatory of Belgium, Ringlaan 3, 1180 Brussels, Belgium; 5 Université Catholique de Louvain, Louvain, Belgium; 6 Universite. Grenoble Alpes, Universite. Savoie Mont Blanc, CNRS, IRD, Universite Gustave Eiffel, ISTerre, 38000 Grenoble, France

**Keywords:** 6-year cycle, Core motions, Magnetic field, Earth’s rotation, Gravity field, Climate

## Abstract

A 6-year cycle has long been recognized to influence the Earth’s rotation, the internal magnetic field and motions in the fluid Earth’s core. Recent observations have revealed that a 6-year cycle also affects the angular momentum of the atmosphere and several climatic parameters, including global mean sea level rise, precipitation, land hydrology, Arctic surface temperature, ocean heat content and natural climate modes. In this review, we first present observational evidences supporting the existence of a 6-year cycle in the Earth system, from its deep interior to the climate system. We then explore potential links between the Earth’s core, mantle and atmosphere that might explain the observations, and investigate various mechanisms that could drive the observed 6-year oscillation throughout the whole Earth system.

## Introduction

Planet Earth is a dynamically active system where all layers from the inner core to the upper atmosphere interact in complex ways across on a wide range of spatiotemporal scales. Over long geological timescales, the interplay between mantle convection, volcanism, tectonic plate distribution, global water cycle, surface rock alteration mechanisms and CO_2_ exchange between the atmosphere and the oceans has kept the Earth’s climate in a state of quasi-equilibrium (called “the tectonic thermostat”) over the Earth history. This equilibrium, along with the presence of a strong internal magnetic field and its protective magnetosphere, shielding the planet from harmful cosmic rays and solar wind, has enabled the development and sustenance of life throughout the Earth’s history (e.g., Langmuir and Broecker, 2012). Global interactions between the deep Earth’s interior, solid layers and surface fluid envelopes have also been reported on timescales of several thousand years, in particular quasi-synchronous variations of the magnetic field and paleoclimate (e.g., Kilifarska et al. 2020; plus many references therein). Links between the geomagnetic field and the atmosphere have also been documented on a century timescale (Cnossen et al. 2016). More recently, a 6-year cycle has been reported in the Earth’s climate (Cazenave et al. 2023; Pfeffer et al. 2023a,b). A similar 6-year oscillation had already been observed in fluid core motions (Gillet et al. 2010, 2022a,b), magnetic field (e.g., Silva et al. 2012, Chuillat and Maus, 2014, Kloss and Finlay 2019), rotation of the Earth’s mantle (or equivalently in the length of day—LOD—e.g., Vondrak and Bursa, 1977; Abarca del Rio et al. 2000 and many subsequent publications), polar motion (Chen et al. 2019), as well as ellipticity of the Earth’s equator and in crustal deformations (Chao and Yu 2020; Watkins et al. 2018; Cheng 2024).

The studies by Cazenave et al. (2023) and Pfeffer et al. (2023b) noted a quasi-synchronicity between the behavior of several global observables, including the magnetic field, gravity field, solid Earth rotation, angular momentum of the atmosphere at this 6-year frequency. They suggested that the entire Earth system is oscillating at this 6-year period, driven by mechanisms that have yet to be identified. In this paper, we review current understanding of the 6-year cycle in the Earth’s rotation and deep interior, as well as the recently observed 6-year cycle in climate parameters. Next, we explore different scenarios and potential mechanisms that could explain the observed 6-year oscillation of the entire Earth system.

It is important to note that the term “6-year cycle” should be considered as generic. As highlighted by Pfeffer et al. (2023b), some dispersion (up to +/- 1 year) may occur around the 6-year period depending on the duration and specifics of the observational records analyzed.

## Review of the 6-Year Cycle in the Earth’s Rotation, Deep Interior, Gravity Field And Crustal Deformations

### Solid Earth Rotation

The rotation rate of the solid Earth, or equivalently the length of day (LOD, which is inversely proportional to the rotation rate), varies over a wide range of timescales, from sub-daily to secular and longer periods (Dickey 1995). These variations, presently observed with high-precision space geodetic techniques, particularly Very Long Baseline Interferometry (VLBI) and Global Navigation Satellite Systems (GNSS), are influenced by numerous astronomical, geophysical, oceanic and atmospheric phenomena (Munk and McDonald, 1960; Lambeck 1980). At periods shorter than 1-3 years, variations of the solid Earth’s rotation are primarily driven by atmospheric effects through the exchange of angular momentum (the property of a rotating body expressed by the product of its moment of inertia by its angular velocity) between the atmosphere and the Earth’s mantle (e.g., Lambeck 1980; Barnes et al. 1983; Dickey 1993; de Viron and Dehant 1999; Gross et al. 2004; Chen 2005; Chao and Yan 2010). At sub-annual, annual and ENSO (El Niño Southern Oscillation) frequencies, climate-related changes in the atmospheric zonal wind circulation, and to a lesser extent redistribution of air masses, transfer angular momentum to the solid Earth via gravitational, friction and pressure (or mountain) torques (i.e., torques resulting from gravitational interactions between the atmosphere and the solid Earth, wind friction on the Earth’s surface and difference in atmospheric pressure on the surface relief), as extensively documented (see Dehant and Matthews 2015 for a review). At such frequencies, the variations in the mantle rotation rate are almost completely explained by the variations in the total angular momentum of the atmosphere which includes both the wind motion and air mass redistribution, the latter contributing only about 10% to the total. This implies that variations of the LOD and total atmospheric angular momentum are in phase, so that their difference cancels out. It is worth noting that the summed contributions of oceanic angular momentum and water mass redistribution on land (land hydrology) are relatively small (e.g., Rosat and Gillet 2023).

Longer-term variations of the Earth’s rotation rate have been observed (e.g., Lambeck 1980), in particular at periods around 18-20 years and 60 years (e.g., Zotov et al. 2016, 2020). The ~18-year variation is partly caused by the 18.6-year lunar solid tide while the multi-decadal evolution (a ~60-year fluctuation has been highlighted, e.g., Roberts et al. 2007) is generally attributed to processes in deep Earth’s interior (via exchange of angular momentum between the solid mantle and the outer fluid core). Although interdecadal oscillations in the atmospheric circulation have been reported (Abarca del Rio et al. 2012), the influence of surface fluid envelopes (atmosphere, oceans and land hydrology) on low-frequency LOD variations is minimal (Rekier et al. 2022).

At the sub-decadal timescale, an oscillation with a period of around 6 years was first reported in early measurements of the Earth’s rotation, resulting in changes in LOD on the order of ~0.1 ms (e.g., Vondrak and Bursa, 1977). Subsequent studies further confirmed the presence of a 6-year cycle in LOD variations (e.g., Abarca del Rio et al. 2000; Gorshkov 2010; Holme and de Viron 2013; Chao et al. 2014; Ding and Chao 2018; Ding 2019; Chen et al. 2019; Duan and Huang 2020; Hsu et al. 2021; Ding et al., 2021; Rekier et al. 2022; Rosat and Gillet 2023).

**Fig. 1:**
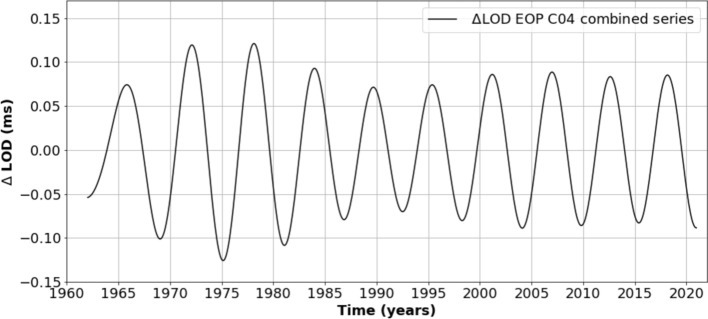
6-year cycle in LOD from 1962 to 2022 (data from the International Earth Rotation and Reference Systems Service—IERS—EOPC04 combined series). The LOD data have been detrended and band-pass-filtered between 5.23 and 7.03 years

Figure 1 illustrates the 6-year oscillation in LOD (the source of data is the EOPC04 combined series from the International Earth Rotation and Reference Systems Service—IERS—Bizouard, 2019). The LOD record, covering the 1962-2022 time span, has been detrended and band-pass-filtered between 5.23 and 7.03 years (see Pfeffer et al. 2023b for details on the band-pass filtering procedure). It is noteworthy that the amplitude of the 6-year cycle, on the order of 0.1 ms, is not perfectly constant with time. It seems more pronounced in the 1970s compared to the 1990s and has remained relatively stable over the past two decades.

As we discuss below, the 6-year cycle in the Earth’s rotation rate is likely linked to processes in the Earth’s core (section 2.3).

### Polar Motion

The Earth’s axis of rotation in a terrestrial reference frame wobbles around the Earth’s axis of inertia predominantly with annual and 14-month (the Chandler wobble) periodicities. It also displays a long-period drift toward Labrador, Canada, at a rate of ~10 cm/year (Lambeck 1980). This long-period drift had generally been attributed to post-glacial rebound (the viscoelastic response of the mantle and crust to the last deglaciation), but a contribution from long-term mass motions due to mantle convection has been also proposed as an additional key driver of this drift (Adhikari et al. 2018; Shahvandi et al. 2024). Chen et al. (2019) identified a 6-year signal in polar motion observations, particularly strong in the eastward component. They attribute this 6-year cycle in the polar motion to deep Earth processes rather than to the redistribution of angular momentum (mass and motion terms) in the surface fluid envelopes. Notably, the 6-year periodicity in the polar motion is close to the beat frequency (of 6.6 years) between the annual polar motion and the Chandler wobble (of period 433 days).

### Core Motions and Internal Magnetic Field

The Earth’s magnetic field has a complex spatial behavior and varies across a wide range of timescales, from years to hundreds of million years. The magnetic field plays a crucial role in shielding the Earth’s surface environment from the charged particles emitted by the Sun and galactic sources. However, this shielding effect is not constant, as the strength and structure of the magnetic field can vary significantly over time. Over long periods, the magnetic field is occasionally disrupted by reversals, during which the field polarity switches to the opposite orientation over a timescale of several thousands of years.

The geomagnetic field is generated within the fluid core through turbulent motions of a liquid iron alloy, driven by heat loss to the above mantle and by the chemical processes involving the mantle and the inner core. While satellite-based and ground observatory data can be used to determine the magnetic field at the top of the core, they alone cannot provide direct insights into the core dynamics. Consequently, our understanding of Earth’s magnetic field, though constrained by the knowledge of its radial component value at the core’s surface, relies on dynamical assumptions about hydromagnetic processes occurring in the core. Even the dynamics near the top of the core have been subject of ongoing debate for decades due to the hill-posed nature of the inverse problem (Holme  2007). A key question concerns the rapid changes in the magnetic field that have been observed during the continuous satellite era (i.e., since 1999). Olsen and Mandea (2008) showed that rapid (interannual) changes in the magnetic field are indicative of resolved transient fluid flows at the top of the core. These flows are spatially localized and involve surprisingly large local magnetic accelerations (see also Gillet et al. 2015, 2022a, b; Kloss and Finlay 2019; Whaler et al. 2022; Ropp and Lesur 2023), known as geomagnetic jerks. These findings suggest that short-term fluctuations in the core’s magnetic field are robust features originating from rapid core dynamics. Such a hypothesis is consistent with advanced numerical simulations of the geodynamo (Aubert and Finlay 2019; Aubert et al. 2022).

Curry (1973), and later on Silva et al. (2012), identified a 6-year periodic signal in the magnetic field record. This periodic signal is dominated by spherical harmonics of degree 1–order 1 and degree 3–order 1. They also estimated the spatial characteristics of the 6-year cycle, finding that the dominant regional patterns are concentrated within a latitudinal band of ~30° width around the equator, primarily in the Atlantic and Indian oceans. These regional patterns closely align with recent estimates of the geometry of the 2003 and 2007 geomagnetic jerks (Olsen and Mandea 2007, 2008; Chulliat and Maus 2014).

**Fig. 2:**
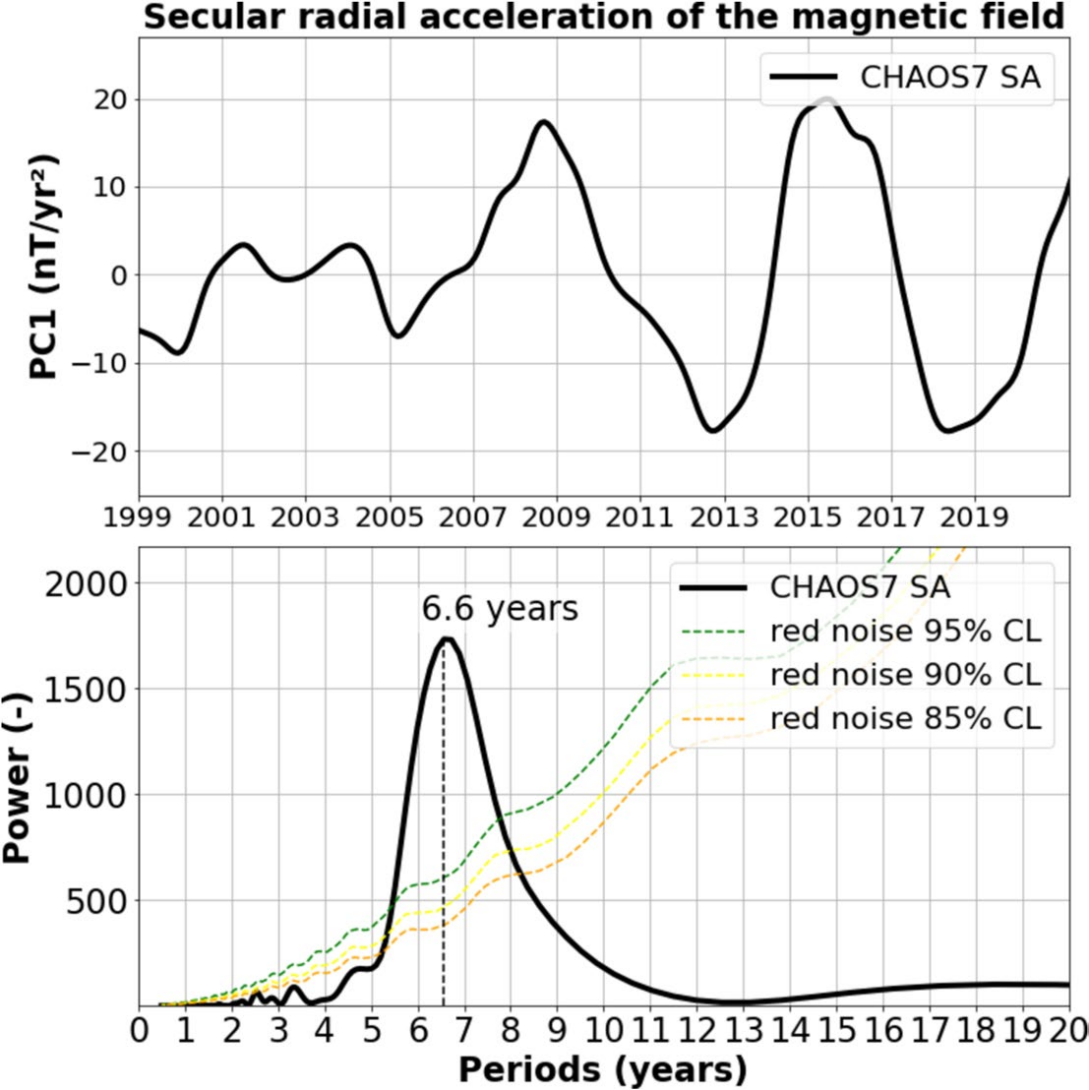
Upper panel: temporal projection for the 1st PC from the EOF decomposition of the magnetic field secular acceleration, based on the CHAOS7 geomagnetic model over 1999–2020. Lower panel: Power spectrum of the 1st PC time series shown in the upper panel. The colored dashed curves represent different confidence levels (adapted from Pfeffer et al. 2023b)

Additional analyses of magnetic field observations confirmed the presence of a 6-year cycle in the secular acceleration (i.e., the second time derivative) of the radial component (Silva et al. 2012; Gillet et al. 2010, 2022a, b; Lesur et al. 2022). This is illustrated in Figure 2 (top), which shows the temporal projection for mode (or principal component, PC) number 1 from the EOF (empirical orthogonal function) decomposition of the secular acceleration observed in the CHAOS7 magnetic field model (Finlay et al. 2020). Figure 2 (bottom) also displays the power spectrum of the PC mode 1, clearly indicating a peak around 6-7 years.

**Fig. 3:**
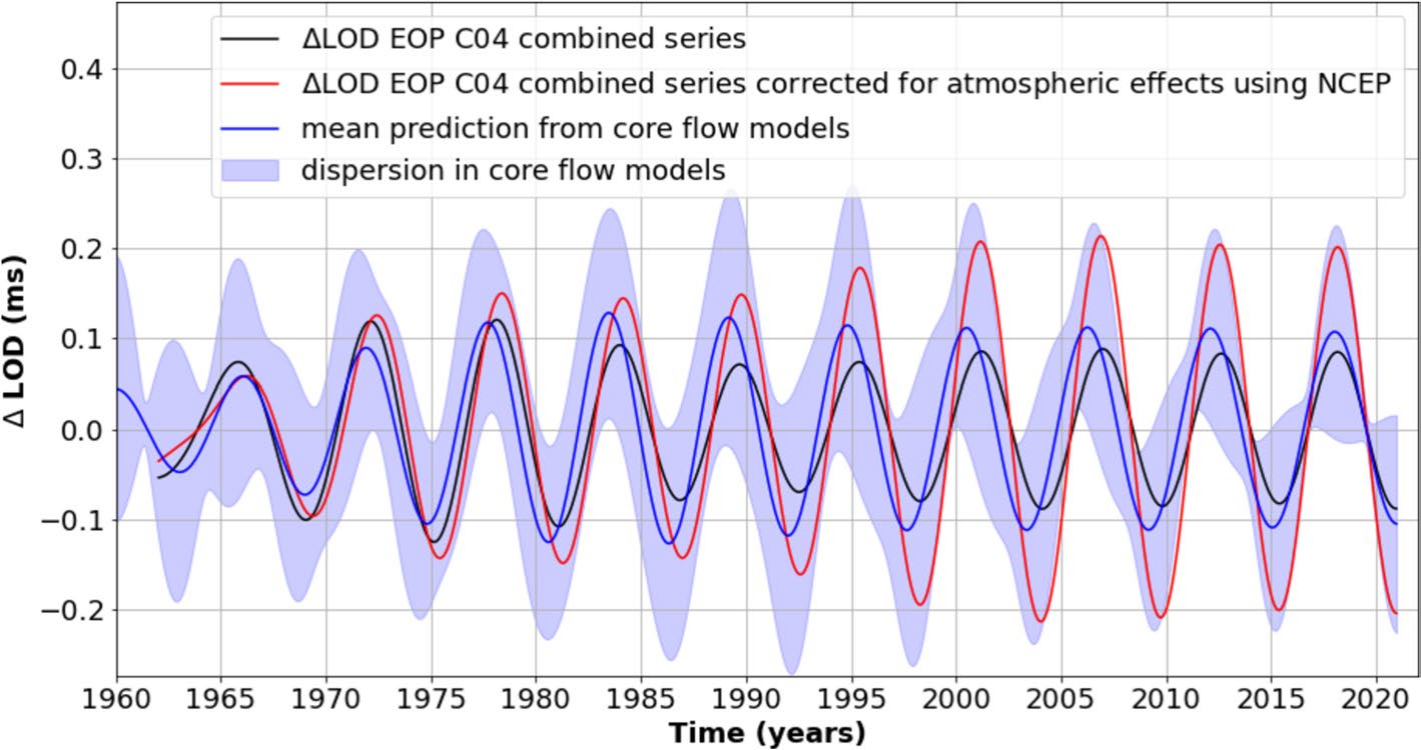
Six-year cycle predicted from an ensemble of 50 estimates of the core angular momentum based on core flow modeling and magnetic data (from Gillet et al. 2022a). The core flow model dispersion (shaded area) is calculated at 1 standard deviation of the model ensemble. The 6-year cycle in LOD is also shown. The black curve is just LOD while the red curve shows LOD corrected for the atmospheric angular momentum (see Sect. 3). The LOD and core angular momentum time series have been band-pass-filtered between 5.23 and 7.03 years

The observed 6-year oscillation in the internal magnetic field is most likely driven by physical processes occurring in the fluid outer core. Analysis of magnetic field data combined with magnetohydrodynamic modeling of the fluid core (Gillet et al. 2010, 2022a,b; Istas et al. 2023) has revealed the existence of torsional Alfven waves and of Magneto-Coriolis waves, both with a periodicity close to 6 years. Fluid motions in the core carry angular momentum that may be transferred to the mantle via core–mantle coupling. Relying on geomagnetic data and on inferred core flow modeling, Gillet et al. (2010) showed that the 6-year signal in LOD can be accurately reproduced by a geostrophic wavelike pattern traveling in the outer core from the inner core to the core–mantle boundary (CMB) equator (see also Istas et al. 2023). Two types of torques have been invoked. The first involves electromagnetic coupling between the fluid core and an electrically conducting mantle (for a recent discussion of this issue, see Schwaiger et al. 2024). The second mechanism is based on a mantle–inner core gravitational coupling (e.g., Chao 2017), in the presence of density anomalies of degree 2 in the deep mantle (see Buffet, 1996). A combination of gravitational coupling between the mantle and the inner core, and electromagnetic coupling has also been proposed by Mound and Buffet (2006) as a potential mechanism for explaining the 6-year LOD variations. Figure 3 shows the 6-year variation observed in LOD (corrected or not for the atmospheric angular momentum; see section 3) alongside the mean core angular momentum at 6 years predicted from 50 core flow models (Gillet et al. 2022a). Although the dispersion (one standard deviation about the mean) of the different core flow models is rather large, the average value agrees well with the observed LOD variations. When LOD is corrected for atmospheric angular momentum, the LOD variation exceeds the mean core angular momentum, but remains within the core angular momentum’s dispersion range.

**Fig. 4:**
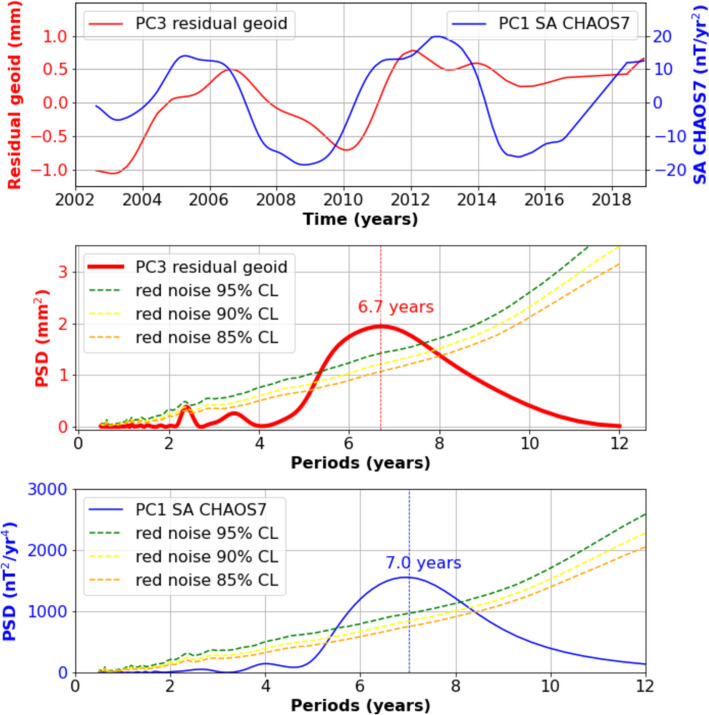
Upper panel: comparison of the temporal projection for PC from an EOF analysis of the CHAOS7 geomagnetic secular acceleration (radial component, 1st PC; blue curve) and geoid anomaly estimated from GRACE and GRACE-FO observations corrected for glacial isostatic adjustment, hydrology and ocean–atmosphere contributions (3rd PC; red curve). Medium panel: Power spectrum of the GRACE-based 3rd PC. Lower panel: Power spectrum of the CHAOS7-based 1st PC. The colored dashed curves in the medium and lower panels represent different confidence levels (adapted from Pfeffer et al. 2023b)

Mandea et al. (2012, 2015) identified a temporal correlation at interannual timescale between the Earth’s magnetic field and gravity field. This correlation was interpreted as an exchange of matter between the fluid core and the lower mantle at the CMB. Additionally, it appeared that this temporal correlation is suggestive of a 6–7-year periodicity (Fig. 4). The discovery of a 6-year periodicity in the global gravity field is a recent finding. Small gravity signals from the deep Earth interior due to mass redistribution in the core or at the CMB could contribute to the observed cycle in the gravity field. However, based on mechanisms (associated with the dynamical pressure at the core surface, or the oscillations of a non-spherical inner core resulting from lateral density variations within the mantle) and observations, Gillet et al. (2021) and Lecomte et al. (2023a,b) placed constraints on the contribution of core dynamics to gravity field variations. They concluded that the potential core signals are still below the uncertainty level of the gravity field observations from the GRACE (Gravity Recovery and Climate Experiment) and GRACE Follow-On (FO) space gravimetry missions, making them difficult to detect (see also Dumberry and Mandea 2021). However, a recent anomalous north–south gravity signal has been detected by Gaugne et al. (2024) across the Atlantic–African continent boundary, starting in January 2007 and evolving over several years. Gaugne et al. (2024) suggest that the signal cannot be fully explained by hydrological or oceanic changes, indicating an origin in the solid Earth. Their modeling points to vertical mass displacements due to the perovskite-to-post-perovskite phase transition, likely caused by thermal anomalies near the base of the African Low Shear Velocity Province. These authors propose that this mechanism may create a dynamic topography at the CMB over a few years.

Meanwhile, mass redistributions within the surface fluid envelopes (atmosphere, oceans, cryosphere and land hydrosphere) seem more likely responsible for the 6-year cycle in the observed gravity field. This will be discussed in Sect. 3.

### Solid Earth Parameters

Using satellite laser ranging (SLR) and GRACE space gravimetry data, Ding and Chao (2018), and Chao and Yu (2020) reported a 6-year variation in the degree 2, order 2 spherical harmonics of the gravity field (or equivalently in the dynamical ellipticity of the Earth’s equator) and attributed it to the gravitational coupling between the inner solid core and the Earth’s mantle.

This finding was confirmed by a recent study by Cheng (2024) who reports a close to 6-year cycle in the ellipticity of the Earth’s equator, using 31-year-long SLR and 22-year-long GRACE/GRACE-FO datasets. Cheng (2024) favors mass redistribution in the surface fluid envelopes to explain this observation.

Another study by Watkins et al. (2018) detected a 6-year cycle in the deformation of the Earth’s crust from the analysis of more than 500 GNSS time series. According to these authors, loading from the surface fluid envelopes (atmospheric, oceanic and hydrological loading) cannot explain such an observation. They rather invoked dynamical pressure coupling at the CMB while inner core–mantle gravitational coupling was also proposed by Ding and Chao (2018). However, further analyses by Rosat et al. (2021) have questioned the significance of such signals, found to be larger than predicted core-induced surface deformation (Gillet et al. 2021). Rosat et al. (2021) suggested that, if real, they likely result from hydrological loading rather than from deep Earth processes.

## The 6-year Oscillation of The Climate System

Several recent studies have identified a 6-year cycle in various climate parameters, including the rate of global mean sea level rise, glacier and Greenland ice mass loss rate, precipitation, land water storage, lake water levels, ocean heat content and Earth energy imbalance, atmospheric angular momentum and zonal wind speed at all altitudes in the troposphere (Moreira et al. 2021; Pfeffer et al. 2021, 2023a,b; Pfeffer et al., 2024). Previous research also reported a ~6-year cycle in the Nile River’s water extrema (Kondrashov et al., 2005; Ghil et al. 2011) as well as in European surface temperature (Jajcay et al. 2016; Meyer and Kantz 2019).

### Sea Level and Components

**Fig. 5:**
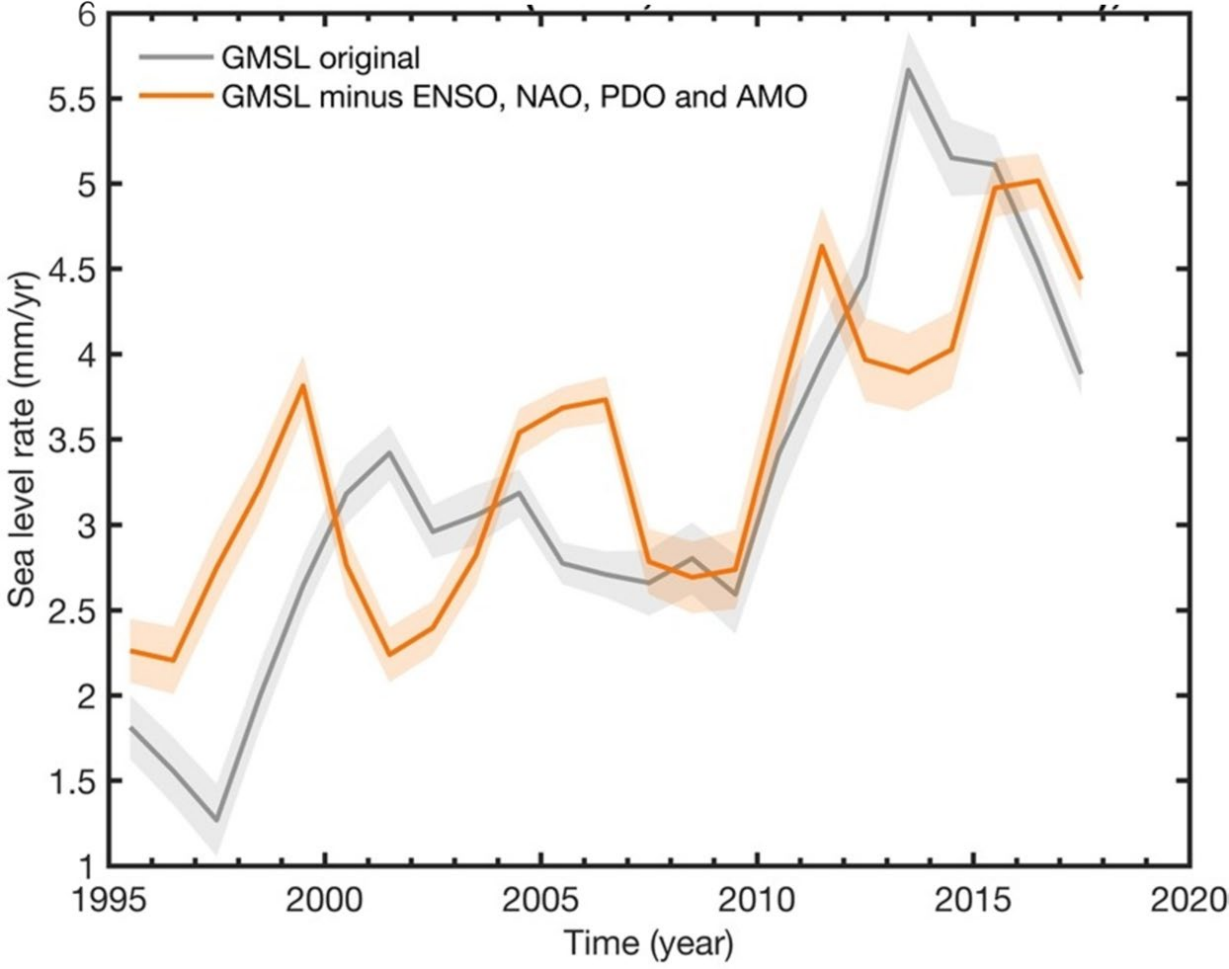
Rate of rise of the altimetry-based global mean sea level (GMSL) over 1993–2019 corrected for the effects of internal climate variability (orange curve). The uncorrected GMSL rate is also shown (gray curve). Shaded areas represent data uncertainty. The internal variability correction is obtained by regressing the GMSL data with the climate indices (ENSO/MEI, NAO, PDO and AMO; details can be found in Moreira et al. 2021). The sea level data are from AVISO (www.aviso.fr) (Figure adapted from Moreira et al. 2021)

Moreira et al. (2021) discovered that the rate of change in the satellite altimetry-based global mean sea level (GMSL) displays a 6-year fluctuation after partly correcting the GMSL time series from the interannual variability related to internal climate modes (Multivariate ENSO Index—MEI, Pacific Decadal Oscillation—PDO, North Atlantic Oscillation—NAO and Atlantic Multi-decadal Oscillation—AMO) (Figure 5). As discussed by Moreira et al. (2021), it is unlikely that this cycle is due to errors in the geophysical corrections applied to the altimetry data. Main contributors to the GMSL variations include ocean thermal expansion due to ocean warming, land ice melt from glaciers, Greenland and Antarctica ice sheets, and terrestrial water storage changes. Moreira et al. (2021) found that the mass components (in particular glaciers and Greenland mass balances, and terrestrial water storage) also display a notable 6-year cycle. Interestingly, a recent study by Bhaskar (2024) revealed that glacial earthquakes related to iceberg calving around Greenland also exhibit a 6-year periodicity.

### Natural Climate Modes

Moreira et al. (2021) also showed that several climate modes (i.e., natural—internal—climate variability) display some energy around 6 years. This is the case for PDO and AMO. Since these indices are based on the combination of various atmospheric and oceanic variables (e.g., atmospheric pressure, sea surface temperature, surface winds, etc.), this clearly suggests that the 6-year cycle influences the climate system.

### Land and Sea Surface Temperature

A ~7-year cycle had been reported in European surface temperature (Jajcay et al. 2016; Meyer and Kantz 2019), potentially linked to the NAO.

**Fig. 6:**
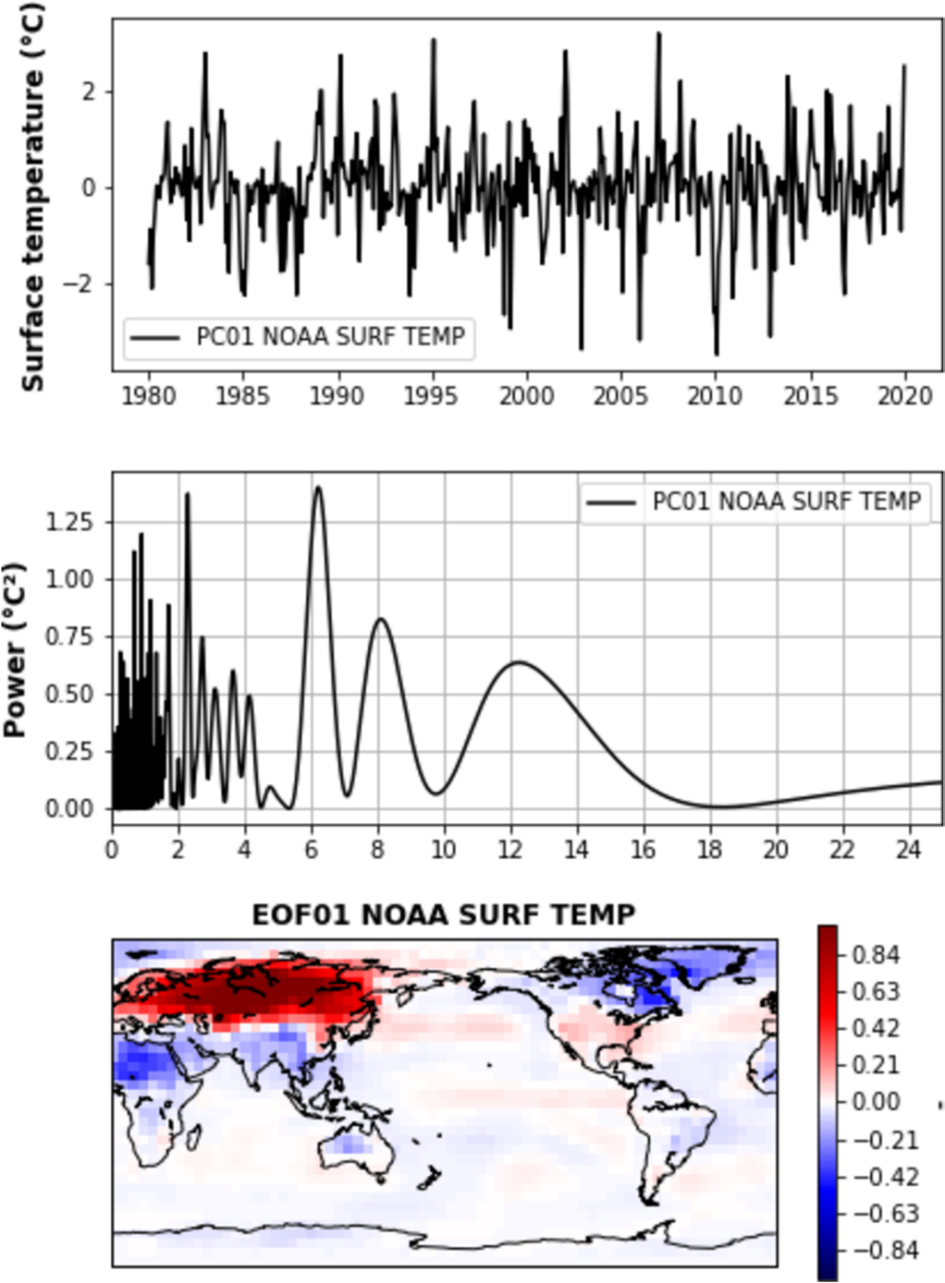
EOF mode 1 of the gridded surface temperature data over 1980–2022 (the data have been de-seasonalized and quadratically detrended). Top panel: Mode 1 principal component (in °C). Medium panel: Power spectrum of the mode 1 principal component (horizontal axis: period in years). Lower panel: Mode 1 spatial patterns (unitless). (Source of data: NOAAGlobTemp dataset)

In our analysis, we examined global 5°x5° gridded surface temperature data using the NOAA (National Oceanic and Atmospheric Administration) merged land- ocean global surface temperature dataset (NOAAGlobalTemp, version 6.0.0) which combines long-term sea surface temperature and land surface temperature datasets (Huang et al. 2024). We removed the seasonal signal and a quadratic trend, and analyzed the data over the 1980-2022 period. Figure 6 presents the first mode of an EOF decomposition of this global Earth’s temperature dataset.

The power spectrum of the mode 1 principal component reveals a dominant 6-year peak. The associated spatial pattern highlights a strong signal over the Arctic regions. It is worth noting that the 6-year signal over Eurasia is in opposition of phase with that over North America and North Atlantic (Greenland region). The other modes of the EOF decomposition appear to correspond to natural climate modes (with mode 2 linked to ENSO and mode 3 to the PDO).

### Global Ocean Heat Content

The global mean ocean heat content (GOHC) reflects the total energy absorbed and stored by oceans. The oceans are the primary reservoir of excess heat due to human activities accumulated in the climate system over the recent decades (e.g., von Schuckmann et al., 2016, 2023; IPCC 2019). GOHC can be estimated by correcting the satellite altimetry-based GMSL for the ocean mass component derived from GRACE/GRACE FO-based space gravimetry measurements (Marti et al. 2022, 2024). The ocean mass-corrected GMSL represents the global mean thermal expansion over the whole ocean depth, from which GOHC can be derived. To extend the GOHC time series prior to the launch of the GRACE mission (in 2002), the ocean mass component can be estimated using a sea level budget approach (Horwath et al. 2022). This ocean mass-corrected GMSL approach also permits to estimate the Earth’s Energy Imbalance (EEI; the difference between incoming solar energy and energy reflected back into space) by computing the time derivative of GOHC (see Marti et al. 2022, 2024 for details). We used the GOHC and EEI products (Magellium/LEGOS, 2023) produced by Magellium/LEGOS and distributed by AVISO+ (https://aviso.altimetry.fr) with support from CNES (Centre National d’Etudes Spatiales).

**Fig. 7:**
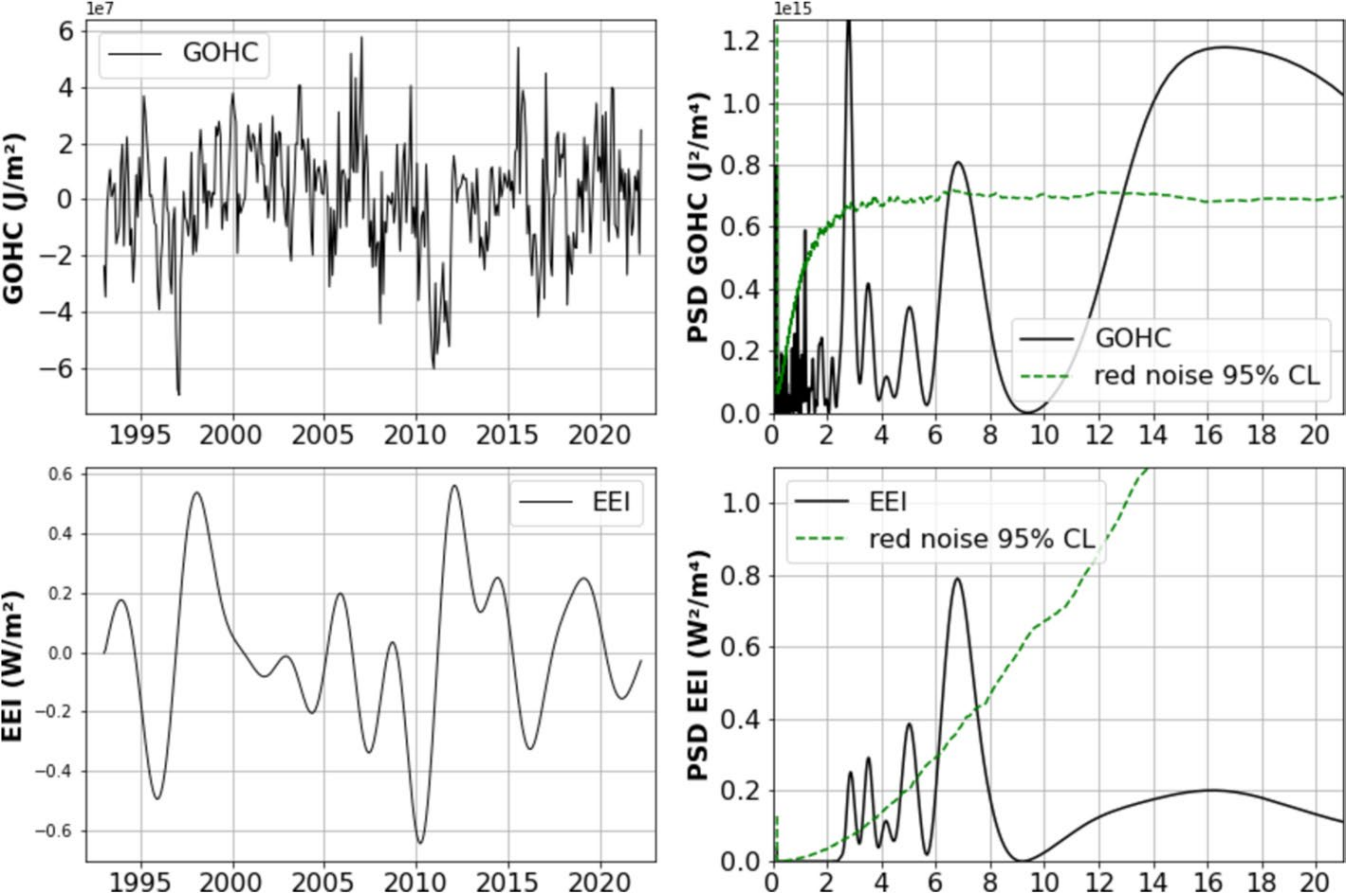
Upper panel: Global ocean heat content (GOHC) time series from 1993 to 2023 (left) and associated power spectrum (right) with the horizontal axis representing periods in years (data derived from altimetry-based global mean sea level corrected for the ocean mass contribution from GRACE/GRACE FO). The bottom panels show the Earth’s energy imbalance (EEI) (left) and its associated power spectrum (right) with the horizontal axis representing periods in years. The green dashed curves in the right-hand side panels represent the confidence level (data from Magellium/LEGOS, 2023)

In addition to a strong positive trend due to anthropogenic global warming, GOHC displays interannual fluctuations due to internal climate variability. Besides an important variation of 2-3-year period related to ENSO, GOHC presents a significant oscillation around 6-7 years as illustrated in Figure 7.

### Land Hydrology and Precipitations

**Fig. 8:**
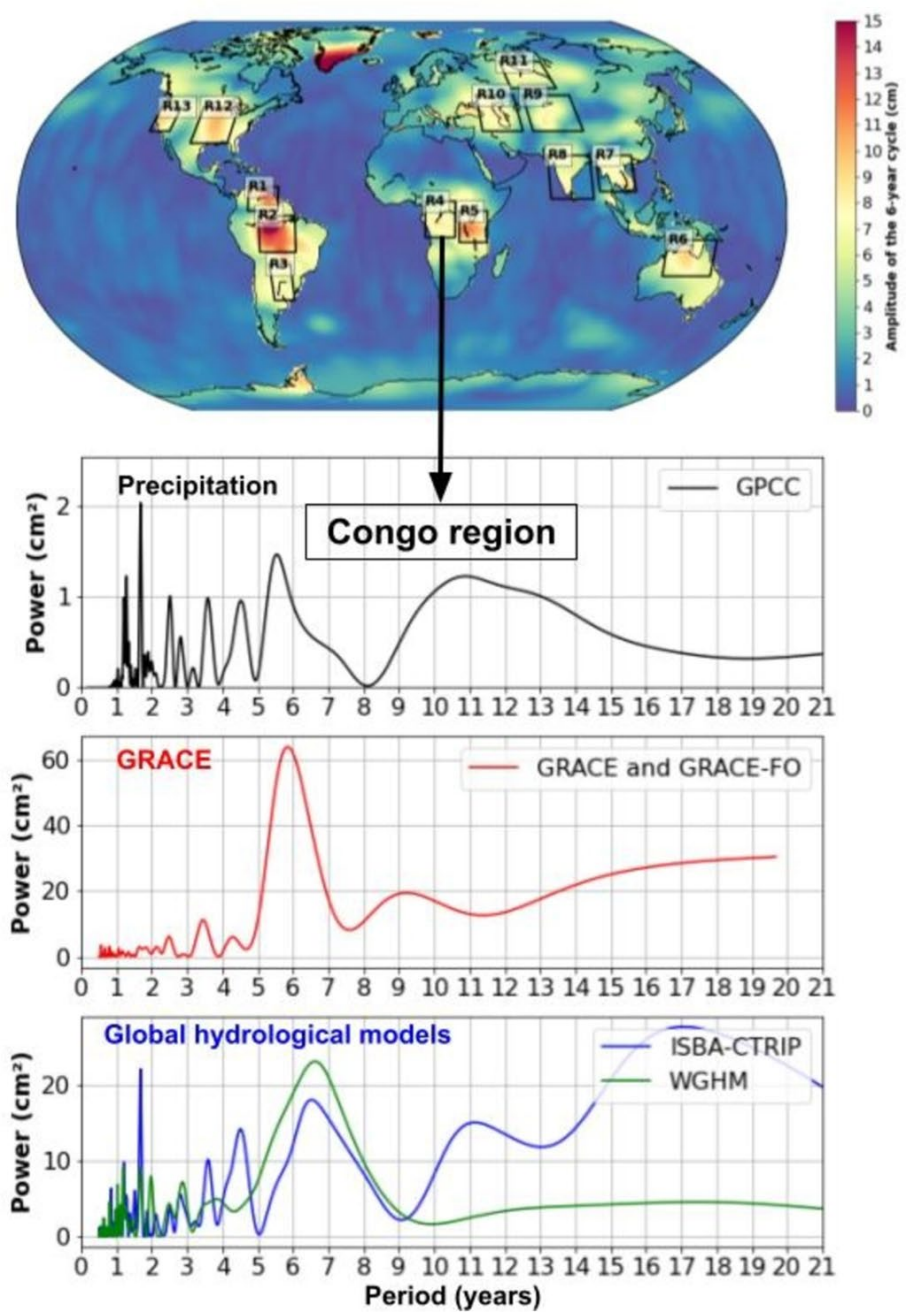
River basins showing a significant 6-year cycle in water storage (squares on the map) and spectral analyses of precipitation (black curve), water storage from GRACE/GRACE-FO (red curve) and from global hydrological models (blue and green curves) in the Congo Basin (adapted from Pfeffer et al. 2023b). Detailed information on the precipitation datasets and hydrological models are provided in Pfeffer et al. (2023b)

Recent studies by Pfeffer et al. (2021, 2023a,b) have identified a 6-year cycle in terrestrial water storage based on space gravimetry missions GRACE and GRACE-FO. These authors also revealed a 6-year cycle in observed precipitation and water storage estimated with global hydrological models. This 6-year cycle is evident across specific river basins (also over large aquifers and large lakes) on all continental areas. The cycle is particularly pronounced in regions such as the Amazon and Orinoco River basins in South America, the Congo and great lakes region in Africa, the Mississippi and Central Valley in North America, and multiple areas across Eurasia. Figure 8 presents a global map of river basins where a 6-year cycle in water storage is significant. It also includes power spectra of precipitation, GRACE-based water storage and water storage estimated from two global hydrological models for the Congo Basin. All three metrics show a clear 6-7-year cycle, reinforcing the consistency of this periodic behavior.

### Atmospheric Angular Momentum

Similar to other climate parameters, the atmospheric angular momentum (AAM) exhibits a 6-year oscillation, with an amplitude in the range 0.07-0.09 ms (when expressed in terms of LOD equivalent) (Pfeffer et al. 2023b). A recent study by Pfeffer et al. (2024) analyzed gridded zonal wind data from 1960 to 2020, using different atmospheric reanalyses, including NCEP (National Centers for Environmental Prediction, Kalnay et al. 1996) and ERA-Interim (European Centre for Medium-Range Weather Forecasts/ECMWF reanalysis, https://www.copernicus.eu/fr/node/67295)*.* The zonal winds (positive eastward) were averaged by longitude across different latitude bands and at different altitudes within the troposphere. This study revealed that tropospheric zonal winds also follow a 6-year oscillation, which remains coherent (i.e., perfectly in phase) across all latitudes and altitudes, from the surface to the tropopause. This suggests that the atmosphere oscillates as a whole at the 6-year frequency.

**Fig. 9:**
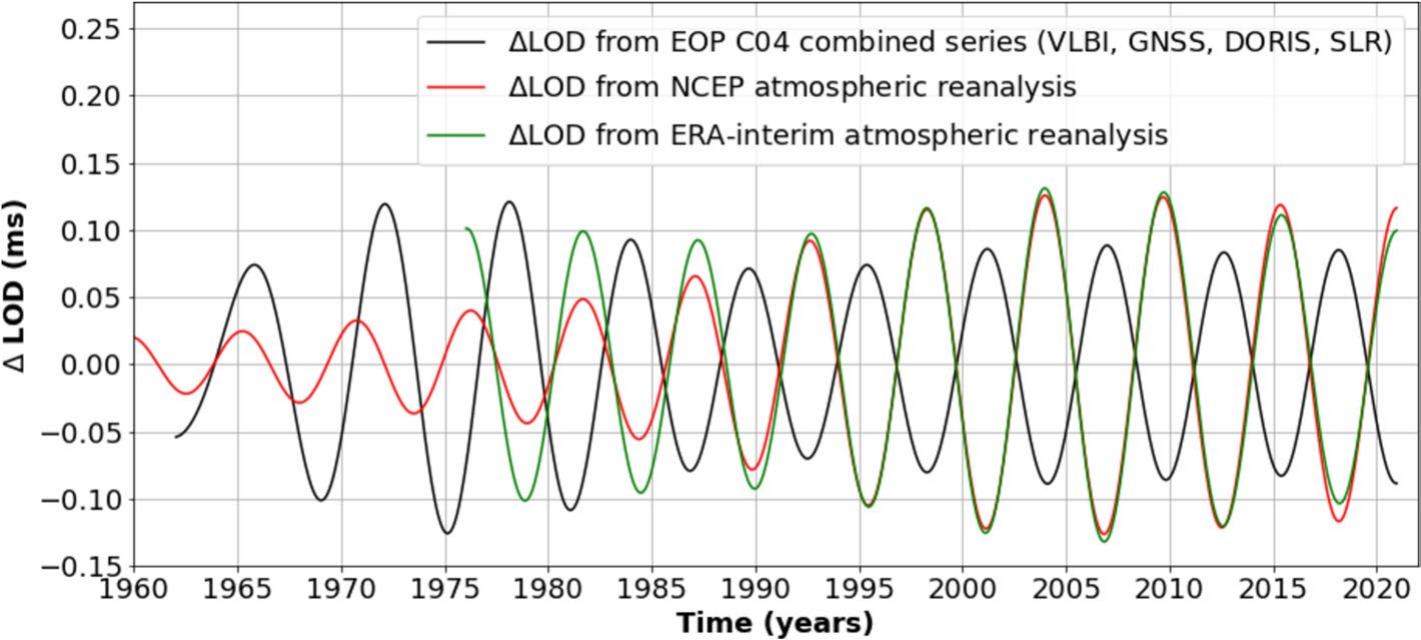
Six-year cycle in the LOD (black curve) from 1960 to 2022 and AAM (expressed in equivalent LOD and noted ΔLOD from NCEP/ERA-Interim in the figure) based on two atmospheric reanalyses (NCEP/NCAR, Kalnay et al. 1996, and ERA-Interim, https://www.copernicus.eu/fr/node/67295) (red and green curves). The LOD and AAM time series have been band-pass-filtered between 5.23 and 7.03 years. Units in ms

An important finding from Pfeffer et al. (2023b, 2024) is that the 6-year cycle in AAM appears to be in opposition of phase with that of the LOD (see Figure 9 showing the 6-year cycle of LOD and AAM covering the period from 1960 to 2022). This is consistent with previous findings by Chen et al. (2019) and Rekier et al. (2022) who noted that correcting LOD for the angular momentum contribution of the surface fluid envelopes (atmosphere, ocean and hydrosphere) did not lead to canceling the LOD 6-year variations (as for the seasonal and ENSO frequencies), but rather enhanced the LOD 6-year cycle (see also Figure 3). However, it is worth noting that the phase opposition between AAM and LOD is not stationary. It is clearly observed from around 1985 onward, but prior to that, a phase shift between the two curves can been seen. In addition, while the NCEP and ERA-Interim data show good agreement from the early 1990s onward, they differ in earlier periods, potentially due to lower wind data quality in the earlier record. Thus, the above-mentioned phase shift may not have a physical origin, but may simply result from less precise wind data before 1990.

The contribution from the core angular momentum, shown by the blue curve in Fig. 3, is in phase with the LOD observations and has a similar amplitude. In contrast, the LOD contribution from the atmosphere, represented by the red/green curves in Fig. 9, is out of phase with the LOD observations.

The conservation of angular momentum is a fundamental property in rotating systems, as long as they are not subject of external torques. Any change in angular momentum in one part of the system must be balanced by an equal and opposite change in the rest of the system. This principle applies to the Earth system at seasonal frequency where change in the atmospheric angular momentum, essentially due to seasonal changes of the tropospheric zonal wind circulation, results in opposite change in the rotation of the solid Earth (i.e., the mantle). This phenomenon was first demonstrated by Munk and MacDonald (1960) and Lambeck (1980) and has been confirmed by many subsequent studies. Additionally, it has been established that transfer of angular momentum from the atmosphere to the solid Earth also occurs at ENSO frequencies (around 2-3 years) (Chao 1984; Dickey et al. 1992; de Viron and Dickey 2014; Lambert et al. 2017; Yu et al. 2021). At the seasonal and ENSO frequencies, ocean and hydrosphere angular momentums also contribute to the transfer of angular momentum to the mantle, though only to a limited extent. The phase opposition between the LOD and AAM at a 6-year period suggests that the Earth’s mantle and the atmosphere oscillate in phase. Since AAM represents air motion relative to the solid Earth, in the eastward direction, this also means that the atmosphere rotates in the same direction as the Earth’s mantle during this 6-year oscillation. Implications on this observation are discussed in Section 4.

### Synchronicity of Earth Global Observables at the 6-Year Frequency

As discussed above, the 6-year cycle influences several global climate-related observables, including atmospheric angular momentum, globally averaged gravity field, magnetic field and rotation of the Earth’s mantle. Notably, these oscillations appear to be quasi-synchronous, as indicated by the extrema in Figures 2, 3, 4 and 9. While correlation does not mean causality, this quasi-synchronicity hints at a possible connection between these global observables.

For other variables, the 6-year cycle is only observed at regional or local scale. This is particularly true for surface temperature (Figure 6) as well as rainfall and terrestrial water storage (Figure 8). The 6-year cycle has been observed in specific river basins, such as the Amazon, Orinoco, Sao Francisco River basins in South America, Congo and Niger basins in Africa, and Mississippi basin in North America (Pfeffer et al. 2023a,b). Hence, evidence of a 6-year cycle in terrestrial water storage has only been brought at local to regional scales. Additionally, for terrestrial water storage and precipitation, the reported 6-year cycles are not in phase across all regions with the timing of the extrema depending on local climatic conditions.

## Which Mechanisms Could Explain A 6-Year Oscillation of The Whole Earth System?

An abundant literature has shown that the 6-year cycle in the solid Earth’s rotation rate (or LOD) is primarily driven by angular momentum transfer from the core to the mantle (see Rekier et al. 2022 for a synthesis). While the exact nature of the torques at play is still debated, a core-linked origin for the 6-year LOD cycle is now well supported by core flow models inverted from geomagnetic data (e.g., Finlay et al. 2023 for a review). Evidence of a 6-year cycle in the climate system is more recent (e.g., Moreira et al. 2021; Cazenave et al. 2023), and shows unequivocally the occurrence of a 6-year cycle in the AAM (e.g., Chen et al. 2019; Rekier et al. 2022) in opposition of phase with LOD (Pfeffer et al. 2023b).

A 6-year cycle in both the core and the atmosphere may be coincidental, with both components influencing Earth’s rotation rate, with a potentially larger impact from the core than the atmosphere. However, this raises an intriguing possibility: Could there be a connection between internal and external geodynamics? What mechanism(s) could be responsible for the observed 6-year oscillation of the entire Earth system?

Several possible scenarios can be considered. Four are proposed below, keeping in mind that other scenarios would also be possible:

1. Core → Mantle rotation → Atmosphere angular momentum → Climate?

2. Atmosphere angular momentum → Mantle rotation → Core motions?

3. Core → Magnetic field → Magnetosphere modulation on incoming cosmic rays and solar wind → Climate?

4. An external mechanism influencing both climate and core dynamics?

Identifying the mechanism(s) behind this 6-year oscillation in climate is important for improving climate simulations and, if necessary, incorporating additional forcing factors into existing models.

In the following sections, we examine each of these four scenarios and discuss which one(s) could be the most plausible.

### Scenario 1: Core → Mantle Rotation → Atmosphere Angular Momentum → Climate?

In scenario 1**,** the 6-year signal is primarily driven by intrinsic fluid motions within the core. The central question is whether the inferred 6-year cycle observed in the mantle rotation could trigger a corresponding 6-year cycle in atmospheric angular momentum and by extension, influence the climate. To a first approximation, the atmosphere as a whole rotates as a solid body with the Earth’s mantle (e.g., Egger et al. 2007; Wallace et al. 2023). Yet, the 6-year cycle identified in zonal wind circulation (Pfeffer et al., 2024) implies air motions relative to the mantle. Therefore, it is challenging to see how the solid Earth’s rotation could directly generate such significant relative motion within the atmosphere.

Coupling mechanisms at the Earth’s surface, such as topographic, viscous or gravitational interactions between the crust and oceans and the atmosphere, might facilitate momentum transfer between the solid Earth and the atmosphere (and vice versa, as explored in scenario 2; e.g., de Viron and Dehant 1999, 2003; Marcus et al. 2010). Additionally, the impact of changes in the Coriolis parameter (due to the 6-year cycle in the mantle rotation) in the atmospheric equations of motion could provide some insight. However, this hypothesis has yet to be fully investigated. Moreover, such an effect would likely be minimal, as the 6-year LOD variations are on the order of a tenth of millisecond, an almost negligible change compared to overall LOD (i.e., a relative change of approximately 10^-9^).

### Scenario 2: Atmosphere Angular Momentum → Mantle Rotation → Core Motions?

Scenario 2 assumes that the 6-year cycle observed in the zonal circulation of the atmosphere transfers angular momentum to the mantle that further influences core motions. However, as discussed in section 3, the observed opposition of phase between LOD and AAM over the 6-year period suggests that a small amount of angular momentum is transferred from the atmosphere to the solid Earth. Consequently, this would imply that the atmosphere has almost no impact on core dynamics via changes in mantle rotation.

However, it is essential to consider that while both the mantle and the atmosphere oscillate in phase over this 6-year period, the observed zonal wind circulation represents additional air motion relative to the rotating solid Earth. During the increasing phase of the cycle, both the mantle and the atmosphere accelerate, accompanied by an additional atmospheric eastward zonal flow. Similarly, in the decreasing phase, both the mantle and the atmosphere decelerate, with an additional eastward zonal flow in the atmosphere in the same direction. This indicates that the atmosphere (i.e., its solid body rotation coupled to the mantle plus its eastward zonal flow) oscillates with a larger amplitude than the mantle at the 6-year period.

Given the gravitational and pressure torques, and air friction at the Earth’s surface, transfer of angular momentum from the atmosphere to the mantle is expected. This transfer would result in a slight decrease in amplitude of the mantle’s rotation (or, equivalently, an increase in LOD) over the entire 6-year cycle. Indeed, this is precisely what we observe when LOD is corrected for AAM effects, as shown in Figure 3. The larger LOD, after being corrected for AAM, is still largely explained by core flows, as discussed in section 2.3.

While this scenario accounts for part of the observations, it does not explain the origin of the 6-year oscillation in AAM nor its connection to climate. These may be driven either by an independent mechanism (e.g., as those explored in scenarios 3 and 4) or by internal climate variability (i.e., variability that is internally generated within the climate system). The latter hypothesis would imply the existence of an internal mode of variability of about 6 years in the climate, excited by the complex dynamical fluctuations within the atmosphere. The fact that well-known internal climate modes such as PDO and AMO, which reflect independent interactions between different components of the climate system, also display a 6-year cycle would favor this hypothesis.

### Scenario 3: Core → Magnetic Field → Magnetosphere Modulation on Incoming Cosmic Rays and Solar Wind → Climate?

Scenario 3 suggests that the 6-year oscillation of the secular variation of the magnetic field (e.g., Soloviev et al. 2017; Gillet et al. 2022b; Finlay et al. 2023) might be accompanied with a similar oscillation in the magnetic field intensity that further influences the characteristics of the magnetosphere and subsequently modulates the flux (intensity and penetration depth) of galactic cosmic rays and energetic solar particles precipitating into the upper atmosphere. The impact of these highly energetic particles (solar wind and galactic cosmic rays) on climate has been widely studied (e.g., Kirkby 2007; Kilifarska et al. 2020; Kilifarska 2021; Kumar et al. 2023; among others), but research has generally focused on timescales longer than 6 years and on the modulation of the flux by the heliosphere and its interaction with the magnetosphere–ionosphere–atmosphere system. While modulation by the internal magnetic field has occasionally been mentioned, it has not been a primary focus.

Various mechanisms (e.g., chemical reactions in the upper atmosphere, in particular on stratospheric ozone, change in the atmospheric electric conductivity, formation of condensation nuclei affecting cloud formation processes, etc.) have been proposed to assess the impact of precipitation of highly energetic particles on the upper atmosphere and, by extension, on the lower atmosphere dynamics and climate. However, there is still no clear consensus on these mechanisms.

It is worth noting that, until recently, solar effects on climate in coupled climate models (e.g., CMIP—Climate Models Intercomparison Project—models) were limited to changes in solar radiation, like for the 11-year solar cycle. Only the most recent CMIP6 models considered in the 6^th^ assessment report of the Intergovernmental Panel on Climate Change (IPCC, 2021) included more comprehensive solar forcing parameters (total solar irradiance, spectral irradiance, energetic particle precipitation including from galactic cosmic rays; Matthes et al. 2017). However, not all climate models account for the effects of cosmic rays, solar protons and medium energy electrons (e.g., Lurton et al. 2020). Moreover, no current models consider modulation by the Earth’s internal magnetic field.

To further investigate scenario 3, it would be valuable to analyze records of cosmogenic radionuclides that reach the Earth’s surface, while considering that they also contain information on the environmental conditions of transportation and sedimentation, in addition to the cosmic ray flux intensity. Interestingly, a 6-year cycle has been detected in analyses of stratospheric ozone records (e.g., Kane 1988). Additionally, direct core magnetic field-driven changes in temperature and zonal wind circulation in the upper atmosphere, which are communicated to the lower atmosphere via various coupling processes, could link the internal Earth’s magnetic field to climate (Cnossen et al. 2016). Dickey et al. (2011) suggested that the correlation observed between core angular momentum, LOD and global mean Earth’s surface temperature at multi-decadal timescales could be related to charged particle fluxes in the upper atmosphere modulated by the internal magnetic field, via, for example, an impact on cloud formation, hence on climate.

### Scenario 4: External Mechanism Causing Changes in Both Climate and Core Dynamics?

Scenario 4 invokes an external effect on climate such as high-frequency Milankovitch astronomical forcing on the Earth orbital and rotational properties, and associated changes in insolation. The low-frequency Milankovitch forcing on the Earth orbital and rotational parameters well explain the glacial/interglacial climates of the Quaternary. This is the so-called theory of paleoclimate (e.g., Berger 1977, 1988, etc.) that relates long-term changes in the incoming solar energy received at the top of the atmosphere (insolation) and its change in space and time, to variations of the Earth’s orbit around the Sun and in its obliquity, in response to the combined gravitational attraction of the Sun, the planets and the Moon. The main parameters involved are the orbital eccentricity, the obliquity of the Earth axis of rotation in space and the climatic precession. Recent studies have shown that in addition to the well-known 41 000-year and 100 000-year Milankovitch cycles causing the large-scale paleoclimate variations of the last million years, high-frequency cycles in the Milankovitch orbital forcing also exist on interannual and decadal timescales (Loutre et al. 1992; Bertrand et al. 2002; Cionco and Soon 2017; Cionco et al. 2021).

Notably, a significant cycle of 5.9 years has been identified, driven by Jupiter’s gravitational influence on Earth’s orbital eccentricity, obliquity and climatic precession (i.e., the position of the perihelion of the Earth’s orbit with respect to the vernal equinox). Loutre et al. (1992) used simplified climate models to estimate that the amplitude of insolation changes at this frequency is ≤ 0.2 Wm^-2^. Although this value is small, especially when feedbacks within the climate system are not considered, it should be compared to the current Earth energy imbalance of anthropogenic origin, which is estimated to be in the range of in the range 0.5-1 Wm^-2^ (von Schuckmann et al., 2016, IPCC 2019).

Recent estimates of present-day EEI based on ocean heat content data and CERES (Clouds and the Earth’s Radiant Energy System) observations of the net radiation flux at the top of the atmosphere (Loeb et al. 2018; https://ceres.larc.nasa.gov/data/) give a value of 0.76 +/- 0.2 Wm^-2^ for the period 2006-2020 (Marti et al. 2022) and 0.48 +/- 0.1 Wm^-2^ for the 1971-2020 time span (von Schuckmann et al. 2023). As discussed in section 3, EEI time series analyses show a 6-7-year cycle of ~0.3 Wm^-2^ amplitude, which is of the same order of magnitude as the multi-decadal changes mentioned above (although not much above their level of uncertainty).

Note that current climate models, such as those used in the CMIP6, do not account for high-frequency Milankovitch orbital forcing on climate (Eyring et al. 2016; Kageyama et al. 2017).

If high-frequency Milankovitch forcing were to be responsible for the observed 6-year oscillation of the climate system, one cannot exclude that the latter be amplified by resonance effects with an eventual internal mode of climate variability of 6 years (see scenario 2). This possibility needs to be further investigated. Nevertheless, it is difficult to see how this could also explain the 6-year cycle observed in the Earth’s core. Legaut (2005) explored the possibility of excitation of waves in the core from variations in the external magnetic dipole, but found that the amount of energy imparted to the core was far less than the energy of observed waves. Therefore, the likelihood of such an external effect influencing both climate and core dynamics is considered quite low.

## Discussion

To improve our understanding of the 6-year cycle affecting the Earth system and its potential causes, scenarios 3 and 4 seem promising in explaining part of the observations. Scenario 1 faces challenges in explaining how mantle rotation could induce relative motion in the atmosphere (e.g., zonal winds), while scenario 2 raises questions about why the entire atmosphere oscillates with a 6-year period.

How can we enhance our understanding of the 6-year cycle affecting the Earth system, and what are the potential applications of this knowledge? To begin, a more comprehensive characterization of the 6-year oscillation within the climate system is essential. This should include an analysis of all parameters involved, the amplitude of variations and their three-dimensional distribution, i.e., at global or regional scale, and changes with altitude for certain atmospheric variables. Employing diverse analytical tools and datasets will be crucial. Beyond the observations noted in section 3, we could explore additional climate parameters, such as evaporation, water vapor content, cloud cover, aerosol coverage and stratospheric ozone levels for the atmospheric component; ocean mass, surface currents and coastal sea levels for the oceanographic component; and land and sea ice, snow cover for the cryosphere; river and lake water levels worldwide; and land cover in anthropogenically less influenced areas.

Furthermore, revisiting natural climate modes, such as ENSO, PDO, NAO and AMO, will be important to clarify their connections with climate variables at regional scales.

A significant task would also involve performing statistical analyses of climate-related extreme events—such as storm surges, rainfall and floods—to determine whether any natural disasters exhibit a 6-year recurrence (Ghil et al. 2011). We could leverage artificial intelligence (AI) methods to identify coupled patterns and causal relationships within the climate data and the broader Earth’s system (Runge et al. 2015, 2019; Shahvandi et al. 2024). Additionally, outputs from coupled climate models (e.g., CMIP6 and its future iterations) should be scrutinized to verify whether historical runs replicate a 6-year cycle in interannual climate variability, focusing on both amplitude and timing, considering versions without anthropogenic forcing. This would help testing the hypothesis of an internally generated 6-year cycle within the climate system. These analyses would provide a clearer global picture of the previously unknown properties of the Earth system.

Regarding scenario 3, analyzing various datasets—such as the flux of energetic particles precipitating in the upper atmosphere from cosmic rays and solar wind, along with cosmogenic radionuclides and stratospheric ozone records—will be beneficial. Concurrently, modeling efforts by experts in magnetosphere–ionosphere–atmosphere physics should be made to explore the mechanisms influencing tropospheric dynamics and, consequently, climate. For scenario 4, updated estimates of the high-frequency Milankovitch forcing at 5.9 years and inferred insolation changes would be valuable to confirm earlier findings by Loutre et al. (1992). Further work is necessary to assess its impact on the interannual climate variability.

It is crucial to emphasize that the magnitudes of the 6-year cycle observed in atmospheric and climate parameters are significant. For instance, the amplitude of the 6-year oscillation in atmospheric angular momentum is approximately 0.08 ms (in equivalent LOD), which is about 25% of the annual cycle (0.34 ms, Gross et al. 2004). In the Congo basin, the amplitude in water storage change over a 6-year period is around 20 km^3^, or one-fifth of the annual variation (~100 km^3^, Crowley et al. 2006; Kitambo et al., 2023). The amplitude of the 6-year cycle in the rate of rise of the global mean sea level is about 2.5 mm/year, closely aligned with the 3.4 mm/year long-term rise observed over the past 30 years during the altimetry era (Cazenave and Moreira 2022). The 6-year cycle of the rate of ice mass loss from the Greenland ice sheet has an amplitude of about 1 mm/year in terms of equivalent sea level (Moreira et al. 2021), a noteworthy figure when compared to the 15-year change of 0.9 mm/year from 2003 to 2016 (Horwath et al. 2022). Additionally, the 6-year cycle in Earth’s energy imbalance, inferred from ocean heat content observations, is estimated at 0.3 W/m^-2^—approximately 50% of the average value over the past 15 years (but as noted above only slightly above the uncertainty level).

A clear description of the 6-year cycle within the climate system, coupled with a robust proposal for a viable mechanism linking it to deep Earth processes and solid Earth rotation, would have numerous applications. For example, it could aid in forecasting the future occurrence of extrema (maxima or minima of the 6-year cycle) for selected climate parameters, including precipitation and water storage in certain regions, river and lake levels in specific basins and coastal sea level changes at selected sites.

In contrast to known natural climate modes that lack strict periodicity and therefore limit precise predictability, the quasi-regular recurrence of the 6-year cycle allows for tentative predictions of extrema. This predictability also holds promise for global hydrological models, coupled climate models and decadal forecasts.

Understanding changes in water storage across global river basins and climate variations over interannual to decadal scales is critical for decision-makers and society, particularly in climate-vulnerable sectors. However, global hydrological models do not always perform well regarding water storage compared to GRACE, particularly on interannual timescales (Pfeffer et al. 2021, 2023a). Testing the assimilation of GRACE data into hydrological models could improve interannual water storage predictions, focusing specifically on the 6-year cycle.

Unlike long-term climate projections based on probabilistic approaches, accurate initialization of both externally forced and internally generated components is crucial for near-term decadal predictions—this has historically posed a significant challenge (e.g., Boer et al., 2016; Smith et al. 2019; O’Kane et al. 2023). Consequently, some climate variables are more predictable than others (e.g., surface temperature is typically more reliable than precipitation). Investigating whether the 6-year cycle is more predictable than other interannual or decadal frequencies, given its quasi-periodic nature, would be of particular interest.

## Conclusion

In this review, we have summarized the current knowledge regarding the 6-year cycle detected in the Earth System, from the deep interior to the upper atmosphere. These observations appear robust and consistent, but the origin of this oscillation across the entire Earth remains unknown.

Our goal was not to provide a definitive explanation, but rather to raise key issues that may help unravel these yet unexplained recurrent patterns.

We proposed four non-exhaustive scenarios to explore the possible causes of this 6-year oscillation:

- Scenario 1 (mantle-driven atmospheric motion) is puzzling, yet not entirely out of the question.
- Scenario 2 (atmospheric angular momentum exchange with the mantle) introduces fascinating questions about the origin of this periodic behavior within the climate system.
- Scenario 3 (magnetic field–cosmic ray and solar wind interaction) and Scenario 4 (high-frequency Milankovitch forcing) are promising and theoretically align with parts of the observations. These scenarios merit deeper investigation, as they may offer valuable insights into the phenomenon.

Further progress toward understanding the mechanisms behind this cycle will require multi-disciplinary research. A collaborative effort involving experts in internal geophysics, geodesy, hydrology, atmospheric sciences, climatology, space physics and astronomy is essential to make significant advances.

We hope this review stimulates interest within the geosciences community and sparks new research initiatives to better understand the intriguing and complex functioning of our planet. By doing so, we can better grasp the interconnections between Earth’s internal dynamics, climate variability and the broader environmental system.

## Conflict of interest

The authors declare they have no conflict of interest.

## References

[CR1] Abarca del Rio R, Gambis D, Salstein DA (2000) Interannual signals in length of day and atmospheric angular momentum. Ann Geophys 18:347–364. 10.1007/s00585-000-0347-9

[CR2] Abarca del Rio R, Gambis D, Salstein DA (2012) Interdecadal oscillations in atmospheric angular momentum variations. J Geodetic Sci 2(1):42–52. 10.2478/v10156-011-0025-8

[CR3] Adhikari S et al (2018) What drives 20th century polar motion? Earth Planet Sci Lett 502:126–132. 10.1016/j.epsl.2018.08.059

[CR4] Aubert J, Finlay CC (2019) Geomagnetic jerks and rapid hydromagnetic waves focusing at Earth’s core surface. Nat Geosci 12(5):393–398. 10.1038/s41561-019-0355-1

[CR5] Aubert J, Livermore PW, Finlay CC, Fournier A, Gillet N (2022) A taxonomy of simulated geomagnetic jerks. Geophys J Int 231(1):650–672. 10.1093/gji/ggac212

[CR6] Barnes, R. T. H., Hide, R., White, A. A., & Wilson, C. A. (1983). Atmospheric angular momentum fluctuations, length-of-day changes and polar motion. Proceedings of the Royal Society of London. A. Mathematical and Physical Sciences, 387(1792), 31–73.

[CR7] Berger A (1977) Support for the astronomical theory of climatic change. Nature 269:44–45. 10.1038/269044a0

[CR8] Berger A (1988) Milankovitch theory and climate. Rev Geophys 26(4):624–657. 10.1029/RG026i004p00624

[CR9] Bertrand C, Loutre MF, Berger A (2002) High frequency variations of the Earth’s orbital parameters and climate change. Geophys Res Lett 29(18):1893. 10.1029/2002GL015622

[CR10] Bhaskar K. (2024). Periodicity in Glacial earthquakes: ENSO teleconnection and effect of Ocean tidal loading, submitted.

[CR11] Bizouard C, Lambert S, Gattano C et al (2019) The IERS EOP 14C04 solution for Earth orientation parameters consistent with ITRF 2014. J Geod 93:621–633. 10.1007/s00190-018-1186-3

[CR12] Boer GJ (2016) The Decadal Climate Prediction Project (DCPP) contribution to CMIP6. Geosci. Model Dev. 9:3751–3777. 10.5194/gmd-9-3751-2016

[CR13] Buffett BA (1996) A mechanism for decade fluctuations in the length of day. Geophys Res Lett 23(25):3803–3806

[CR14] Cazenave A, Moreira L (2022) Contemporary sea level changes from global to local scales: a review. Proc Royal Society 478:20220049. 10.1098/rspa.2022.0049 10.1098/rspa.2022.0049 PMC9116442 35645600

[CR15] Cazenave A, Pfeffer J, Mandea M, Dehant V (2023) ESD Ideas: A 6-year oscillation in the whole Earth system? Earth System Dynamics 14:733–735. 10.5194/esd-14-733-2023

[CR16] Chao BF (1984) Interannual length-of-day variation with relation to the southern oscillation/El Nino. Geophys Res Lett 11(5):541–544

[CR17] Chao BF (2017) Dynamics of the inner core wobble under mantle-inner core gravitational interactions. Journal of Geophysical Research: Solid Earth 122(9):7437–7448. 10.1002/2017JB014405

[CR18] Chao BF, Yan H (2010) Relationship between length-of-day variations and angular momentum of geophysical fluids. J Geophys Res 115:B10417. 10.1029/2009JB007024

[CR19] Chao BF, Yu Y (2020) Variation of the equatorial moments of inertia associated with a 6-year westward rotary motion in the Earth. Earth Planet Sci Lett 542:116316. 10.1016/j.epsl.2020.116316

[CR20] Chao BF, Chung W, Shih Z, Hsieh Y (2014) Earth’s rotation variations: a wavelet analysis. Terra Nova 26(4):260–264. 10.1111/ter.12094

[CR21] Chen J (2005) Global mass balance and the length-of-day variations. J Geophys Res 110:B08404. 10.1029/2004JB003474

[CR22] Chen J, Wilson CR, Kuang W, Chao BF (2019) Interannual oscillations in Earth rotation. Journal of Geophysical Research: Solid Earth 124:13404–13414. 10.1029/2019JB018541

[CR23] Cheng M (2024) Decadal Variations in Equatorial Ellipticity and Principal Axis of the Earth from Satellite Laser Ranging/GRACE. Surv Geophys. 10.1007/s10712-024-09852-w

[CR24] Chulliat A, Maus S (2014) Geomagnetic secular acceleration, jerks, and a localized standing wave at the core surface from 2000 to 2010. J Geophys Res Solid Earth 119:1531–1543. 10.1002/2013JB010604

[CR25] Cionco RG, Soon W (2017) Short-term orbital forcing: A quasi-review and a reappraisal of realistic boundary conditions for climate modeling. Earth Sci Rev 166:206–222. 10.1016/j.earscirev.2017.01.01z

[CR26] Cionco RG, Kudryavtsev SM, W. W.‐H. Soon, (2021) Possible Origin of Some Periodicities Detected in Solar‐Terrestrial Studies: Earth’s Orbital Movements. Earth and Space Science. 10.1029/2021EA001805

[CR27] Cnossen I, Liu H, Lu H (2016) The whole atmosphere response to changes in the Earth’s magnetic field from 1900 to 2000: an example of top-down vertical coupling. J Geophys Res Atmos 121:7781–7800. 10.1002/2016JD024890

[CR28] Crowley JW, Mitrovica JX et al (2006) Land Water Storage within the Congo Basin Inferred from GRACE Satellite Gravity Data. Geophys Res Lett 33:19. 10.1029/2006gl027070

[CR29] Currie RG (1973) Geomagnetic line spectra -2 to 70 years. Astrophys Space Sci 21:425–438

[CR30] de Viron O, Dehant V (1999) Earth’s rotation and high frequency angular momentum budget of the atmosphere. Surv Geophys 20:441–462. 10.1023/A:1006723924421

[CR31] de Viron O, Dehant V (2003) Tests on the validity of atmospheric torques on Earth computed from atmospheric model outputs. J Geophys Res B 2(108):2068. 10.1029/2001JB001196

[CR32] de Viron O, Dickey JO (2014) The two types of El-Niño and their impacts on the length of day. Geophys Res Lett 41:3407–3412. 10.1002/2014GL059948

[CR33] Dehant V. and Matthews P.M. (2015) Precession, Nutation and Wobble of the Earth, 552 Pages, Cambridge University Press, ISBN: 1–10746582–610746582–6.

[CR34] Dickey J, MarcusHide S (1992) R. Global propagation of interannual fluctuations in atmospheric angular momentum. Nature 357:484–488. 10.1038/357484a0

[CR35] Dickey JO, Marcus SL, de Viron O (2011) Air temperature and anthropogenic forcing: insights from the solid Earth. J Clim 24:569–574

[CR36] Dickey, J. O., (1993). Atmospheric Excitation of the Earth’s Rotation: Progress and Prospects via Space Geodesy, in Contributions of Space Geodesy to Geodynamics: Earth Dynamics, Geodyn. Ser., vol. 24, edited by D. E. Smith and D. L. Turcotte, pp. 55–70, AGU, Washington, D. C.

[CR37] Dickey J.O. (1995). Earth rotation variations from hours to centuries. In Highlights of Astronomy, Appenzeller (ed.), Cambridge University Press, Vol. 10, 17–44. Printed in the Netherlands. 10.1017/S1539299600010339.

[CR38] Ding H (2019) Attenuation and excitation of the– 6 year oscillation in the length-of-day variation. Earth Planet Sci Lett 507:131–139. 10.1016/j.epsl.2018.12.003

[CR39] Ding H, Chao BF (2018) A 6-year westward rotary motion in the Earth: Detection and possible MICG coupling mechanism. Earth and Planetary Science Letters 495:50–55. 10.1016/j.epsl.2018.05.009

[CR40] Duan P, Huang C (2020) Intradecadal variations in length of day and their correspondence with geomagnetic jerks. Nat Commun 11:2273. 10.1038/s41467-020-16109-8 32385238 10.1038/s41467-020-16109-8 PMC7210880

[CR41] Dumberry M, Mandea M (2021) Gravity variations and ground deformations resulting from core dynamics. Surv Geophys 43(1):5–39. 10.1007/s10712-021-09656-2 35535256 10.1007/s10712-021-09656-2 PMC9050810

[CR42] Egger J, Weickmann K, Hoinka K-P (2007) Angular momentum in the global atmospheric circulation. Rev Geophys 45:RG4007. 10.1029/2006RG000213

[CR43] Eyring V, Bony S, Meehl GA, Senior CA et al (2016) Overview of the Coupled Model Intercomparison Project Phase 6 (CMIP6) experimental design and organization. Geosci Model Dev 9:1937–1958. 10.5194/gmd-9-1937-2016

[CR44] Finlay et al (2020) The CHAOS-7 geomagnetic feld model and observed changes in the South Atlantic Anomaly. Earth Planets Space 72:156. 10.1186/s40623-020-01252-9 10.1186/s40623-020-01252-9 PMC7578192 33122959

[CR45] Finlay CC, Gillet N, Aubert J, Livermore PW, Jault D (2023) Gyres, jets and waves in the Earth’s core. Nature Review Earth & Environment. 10.1038/s43017-023-00425-w

[CR46] Gaugne C, Pannet I, Greff-Lefftz M, Mandea M, Rosat S (2024) Rapid mass changes at the core-mantle boundary originating from the deep mantle as seen by GRACE mission, presentation at the 2024 AGU Fall Meeting, DI12A-06.

[CR47] Ghil M et al (2011) Extreme events: dynamics, statistics and prediction. Nonli Process Geophys 18:295–350. 10.5194/npg-18-295-2011

[CR48] Gillet N, Jault D, Canet E et al (2010) Fast torsional waves and strong magnetic field within the Earth’s core. Nature 465:74–77. 10.1038/nature09010 20445627 10.1038/nature09010

[CR49] Gillet N, Jault D, Finlay CC (2015) Planetary gyre, time-dependent eddies, torsional waves, and equatorial jets at the Earth’s core surface. J Geophys Res: Solid Earth 120(6):3991–4013. 10.1002/2014JB011786

[CR50] Gillet N, Dumberry M, Rosat S (2021) The limited contribution from outer core dynamics to global deformations at the Earth’s surface. Geophys J Int 224(1):216–229. 10.1093/gji/ggaa448

[CR51] Gillet N, Gerick F, Jault D, Schwaiger T, Aubert J, Istas M (2022a) Satellite magnetic data reveal interannual waves in Earth’s core. Proc Natl Acad Sci 119(13):e2115258119. 10.1073/pnas.2115258119 35312364 10.1073/pnas.2115258119 PMC9060525

[CR52] Gillet N, Gerick F, Angappan R et al (2022b) A Dynamical Prospective on Interannual Geomagnetic Field Changes. Surv Geophys 43:71–105. 10.1007/s10712-021-09664-2

[CR53] Gorshkov VL (2010) Study of the interannual variations of the Earth’s rotation. Sol Syst Res 44:487–497. 10.1134/S003809461006002X

[CR54] Gross RS, Fukumori I, Menemenlis D, Gegout P (2004) Atmospheric and oceanic excitation of length-of-day variations during 1980–2000. J Geophys Res 109:B01406. 10.1029/2003JB002432

[CR55] Holme R, de Viron O (2013) Characterization and implications of intradecadal variations in length of day. Nature 499(7457):202–204. 10.1038/nature12282 23846659 10.1038/nature12282

[CR56] Holme R (2007) Large-scale flow in the core. Treatise on geophysics 8:107–130. Ed. P Olson and G Schubert

[CR57] Horwath M, Gutknetch B, Cazenave A et al (2022) Global sea level budget and ocean mass budget, with focus on advanced data products and uncertainty characterization. Earth Syst Sci Data 14:411–447. 10.5194/essd-14-411-2022

[CR58] Hsu CC, Duan PS, Xu XQ, Zhou YH, Huang CL (2021) On the– 7 year periodic signal in length of day from a frequency domain stepwise regression method. J Geodesy 95(5):55. 10.1007/s00190-021-01503-x

[CR59] Huang, B., X. Yin, M. J. Menne, R. Vose, and H. Zhang (2024). NOAA Global Surface Temperature Dataset (NOAAGlobalTemp), Version 6.0.0 . NOAA National Centers for Environmental Information. 10.25921/rzxg-p717.

[CR60] IPCC (2019). IPCC Special Report on the Ocean and Cryosphere in a Changing Climate [H.-O. Pörtner, D.C. Roberts, V. Masson-Delmotte, P. Zhai, M. Tignor, E. Poloczanska, K. Mintenbeck, A. Alegría, M. Nicolai, A. Okem, J. Petzold, B. Rama, N.M. Weyer (eds.)].

[CR61] Istas M, Gillet N, Finlay CC, Hammer MD, Huder L (2023) Transient core surface dynamics from ground and satellite geomagnetic data. Geophys J Int 233(3):1890–1915. 10.1093/gji/ggad039

[CR62] Jajcay N, Hlinka J, Kravtsov S, Tsonis AA, Paluš M (2016) Time scales of the European surface air temperature variability: The role of the 7–8 year cycle. Geophys Res Lett 43(2):902–909. 10.1002/2015GL067325

[CR63] Kageyama M et al (2017) The PMI4 contribution to CMIP4-Part 4: scientific objectives and experimental design of the PMIP4-CMIP6 Last Glacial Maximum experiments and PMIP4 sensitivity experiments. Geosci Model Dev 10:4035–4055. 10.5194/gmd-10-4035-2017

[CR64] Kalnay et al (1996) The NCEP/NCAR 40-year reanalysis project. Bull Amer Meteor Soc 77:437–470

[CR65] Kane RP (1988) Long-term Variation of Total Ozone. Pure Appl Geophys 127:143–154. 10.1007/BF00878695

[CR66] Kilifarska N (2021) Impact of decadal variations of galactic cosmic rays in Earth’s climate variability. Bulg J Phys 47:4–13

[CR67] Kilifarska N, Bakhmutov VG, Melnyk GV (2020) The hidden link between Earth’s magnetic field and climate, ISBN:978-0-12-819346-4. Elsevier, Amsterdam, The Netherlands

[CR68] Kirkby J (2007) Cosmic rays and climate. Surv Geophys 28:333–375. 10.1007/s10712-008-9030-6

[CR75] Kitambo et al (2023) A long-term monthly surface water storage dataset for the Congo basin from 1992 to 2015. Earth Syst Sci Data 15:2957–2982. 10.5194/essd-15-2957-2023

[CR69] Kloss C, Finlay CC (2019) Time-dependent low-latitude core flow and geomagnetic field acceleration pulses. Geophys J Int 217(1):140–168. 10.1093/gji/ggy545

[CR70] Kondrashow D, Feliks Y, Ghil M (2005) Oscillatory modes of extended Nile River records (A.D. 622–1922). Geophys Res Lett 32:L10702. 10.1029/2004GL022156

[CR71] Kumar V et al (2023) The influence of solar-modulated regional circulations and galactic cosmic rays on global cloud distribution. Sci Rep 13:3707. 10.1038/s41598-023-30447-9 36878955 10.1038/s41598-023-30447-9 PMC9988889

[CR72] Lambeck, K. (1980) The Earth’s Variable Rotation: Geophysical Causes and Consequences. 449 pages, Cambridge University Press.

[CR73] Lambert SB, Marcus SL, de Viron O (2017) Atmospheric torques and Earth’s rotation: what drove the milli-second length of day response to the 2015–2016 El Nino?. Earth Syst Dyn 8:1009–1017. 10.5194/esd-8-1009-2017

[CR74] Langmuir CH, Broeker W (2012) How to Build a Habitable Planet: The Story of Earth from the Big Bang to Humankind, 737 pages, ISBN: 9781400841974. Princeton University Press

[CR76] Lecomte H, Rosat S, Mandea M, Boy JP, Pfeffer J (2023a) Uncertainty of Low‐Degree Space Gravimetry Observations: Surface Processes Versus Earth’s Core Signal. J Geophys Res Solid Earth 128(7):e2023JB026503. 10.1029/2023JB026503

[CR77] Lecomte H, Rosat S, Mandea M, Dumberry M (2023b) Gravitational constraints on the Earth’s inner core differential rotation. Geophys Res Lett 50(23):e2023GL104790. 10.1029/2023GL104790

[CR78] Légaut G (2005) *Ondes de torsion dans le noyau terrestre*. Thèse de doctorat. Université Joseph-Fourier-Grenoble I

[CR79] Lesur V, Gillet N, Hammer MD et al (2022) Rapid Variations of Earth’s Core Magnetic Field. Surv Geophys 43:41–69. 10.1007/s10712-021-09662-

[CR80] Loeb NG, Doelling DR, Wang H, Su W, Nguyen C, Corbett JG, Liang L, Mitrescu C, Rose FG, Kato S (2018) Clouds and the Earth’s Radiant Energy System (CERES) Energy Balanced and Filled (EBAF) Top-of-Atmosphere (TOA) Edition-4.0 Data Product. J Climate 31:895–918. 10.1175/JCLI-D-17-0208.1

[CR81] Loutre MF, Berger A, Bretagnon P, Blanc PL (1992) Astronomical frequencies for climate research at the decadal to century time scale. Clim Dyn 7:181–194. 10.1007/BF00206860

[CR82] Lurton T et al (2020) Implementation of the CMIP6 forcing data in the IPSL-CM6A-LR model. J Advances in Modeling Earth Systems 12:e2019MS001940. 10.1029/2019MS001940

[CR83] Magellium/LEGOS. (2023). OHC/EEI from space : Climate indicators: Ocean heat content and Earth energy imbalance (Version V5.0, p. 19 Mo) [Application/x-netcdf]. CNES. 10.24400/527896/A01-2020.003

[CR84] Mandea M, Panet I, Lesur V, De Viron O, Diament M, Le Mouël JL (2012) Recent changes of the Earth’s core derived from satellite observations of magnetic and gravity fields. Proc Natl Acad Sci 109(47):19129–19133. 10.1073/pnas.1207346109 23064635 10.1073/pnas.1207346109 PMC3511155

[CR85] Mandea M, Narteau C, Panet I, Le Mouël JL (2015) Gravimetric and magnetic anomalies produced by dissolution-crystallization at the core-mantle boundary. Journal of Geophysical Research: Solid Earth 120(9):5983–6000. 10.1002/2015JB012048

[CR86] Marcus SL, de Viron O, Dickey JO (2010) Interannual atmospheric torque and El Niño-Southern Oscillation: Where is the polar motion signal? J Geophys Res 115:B12409. 10.1029/2010JB007524

[CR87] Marti F, Blazquez A, Meyssignac B et al (2022) Monitoring the ocean heat content change and the Earth energy imbalance from space altimetry and space gravimetry. Earth Syst Sci Data 14:229–249. 10.5194/essd-14-229-2022

[CR88] Marti, F., Meyssignac, B., Rousseau, V., Ablain, M., Fraudeau, R., Blazquez, A., and Fourest, S. (2024). Monitoring global ocean heat content from space geodetic observations to estimate the Earth energy imbalance, in: 8th edition of the Copernicus Ocean State Report (OSR8), edited by: von Schuckmann, K., Moreira, L., Grégoire, M., Marcos, M., Staneva, J., Brasseur, P., Garric, G., Lionello, P., Karstensen, J., and Neukermans, G., Copernicus Publications, State Planet, 4-osr8, 3, 10.5194/sp-4-osr8-3-2024.

[CR89] Matthes K et al (2017) Solar forcing for CMIP6 (v3.2). Geosci Model Dev 10(6):2247–2302. 10.5194/gmd-10-2247-2017

[CR90] Meyer PG, Kantz H (2019) A simple decomposition of European temperature variability capturing the variance from days to a decade. Clim Dyn 53:6909–6917. 10.1007/s00382-019-04965-0

[CR91] Moreira L, Cazenave A, Palanisamy H (2021) Influence of interannual variability in estimating the rate and acceleration of present-day global mean sea level. Global Planet Change 199:103450. 10.1016/j.gloplacha.2021.103450

[CR92] Mound JE, Buffett BA (2006) Detection of a gravitational oscillation in length-of-day. Earth Planet Sci Lett 243(3–4):383–389. 10.1016/j.epsl.2006.01.043

[CR93] Munk, W. H. and MacDonald, G. J. F. (1960) The Rotation of the Earth. Cambridge University Press, London, 323 pages.

[CR94] O’Kane TJ, Scaife AA, Kushnir Y et al (2023) Recent applications and potential of near-term (interannual to decadal) climate predictions. Front Clim 5:1121626. 10.3389/fclim.2023.1121626

[CR95] Olsen N, Mandea M (2007) Investigation of a secular variation impulse using satellite data: The 2003 geomagnetic jerk. Earth Planet Sci Lett 255:94–105. 10.1016/j.epsl.2006.12.008

[CR96] Olsen N, Mandea M (2008) Rapidly changing flows in the Earth’s core. Nat Geosci 1:390–394. 10.1038/ngeo203

[CR97] Pfeffer J, Cazenave A, Barnoud A (2021) Analysis of the interannual variability in satellite gravity solutions: impact of climate modes on water mass displacements across continents and oceans. Clim Dyn. 10.1007/s00382-021-05953

[CR98] Pfeffer J, Cazenave A, Blazquez A, Decharme B, Munier S, Barnoud A (2023a) Assessment of pluri-annual and decadal changes in terrestrial water storage predicted by global hydrological models in comparison with the GRACE satellite gravity mission. Hydrol Earth Syst Sci 27:3743–3768. 10.5194/hess-27-3743-2023

[CR99] Pfeffer J, Cazenave A, Rosat S, Moreira L, Mandea M, Dehant V (2023b) A 6-Year Cycle in the Earth System. Global Planet Change 229:104245. 10.1016/j.gloplacha.2023.104245

[CR100] Pfeffer J et al (2024) A 6-year cycle in the atmosphere. Geophys Res Lett submitted

[CR101] Rekier J, Chao BF, Chen J et al (2022) Earth’s Rotation: Observations and Relation to Deep Interior. Surv Geophys 43:149–175. 10.1007/s10712-021-09669-x

[CR102] Roberts PH, Yu ZJ, Russell CT (2007) On the 60-year signal from the core. Geophys Astrophys Fluid Dyn 101(1):11–35

[CR103] Ropp G, Lesur V (2023) Mid-latitude and equatorial core surface flow variations derived from observatory and satellite magnetic data. Geophys J Int 234(2):1191–1204. 10.1093/gji/ggad113

[CR104] Rosat S, Gillet N (2023) Intradecadal variations in length of day: Coherence with models of the Earth’s core dynamics. Phys Earth Planet Int 341:107053. 10.1016/j.pepi.2023.107053

[CR105] Rosat S, Gillet N, Boy JP, Couhert A, Dumberry M (2021) Interannual variations of degree 2 from geodetic observations and surface processes. Geophys J Int 225(1):200–221. 10.1093/gji/ggaa590

[CR106] Runge J, Petoukhov V, Donges J et al (2015) Identifying causal gateways and mediators in complex spatio-temporal systems. Nat Commun 6:8502. 10.1038/ncomms9502 26443010 10.1038/ncomms9502 PMC4633716

[CR107] Runge J, Bathiany S, Bollt E-V, G. et al (2019) Inferring causation from time series in Earth system sciences. Nat Commun 10:2553. 10.1038/s41467-019-10105-3 31201306 10.1038/s41467-019-10105-3 PMC6572812

[CR108] von Schuckmann K et al (2023) Heat stored in the Earth system 1960–2020: Where does the energy go? Earth Syst Sci Data 15(4):1675–1709. 10.5194/essd-15-1675-2023

[CR109] Schwaiger T, Gillet N, Jault D, Istas M, Mandea M (2024) Wave-like motions and torques in Earth’s core as inferred from geomagnetic data: A synthetic study. Phys Earth Planet Int 346:107104. 10.1016/j.pepi.2023.107104

[CR110] Shahvandi MK, Adhikari S, Dumberry M et al (2024) Contributions of core, mantle and climatological processes to Earth’s polar motion. Nature Geosciences 17:705–710. 10.1038/s41561-024-01478-2

[CR111] Silva L, Jackson L, Mound J (2012) Assessing the importance and expression of the 6-year geomagnetic oscillation. J Geophys Res 117:B10101. 10.1029/2012JB009405

[CR112] Smith D.M. et al., (2019). Robust skill of decadal climate predictions, *Climate and Atmospheric Science*, 2, 13 ; 10.1038/s41612-019-0071-y.

[CR113] Soloviev A, Chulliat A, Bogoutdinov S (2017) Detection of secular acceleration pulses from magnetic observatory data. Phys Earth Planet Inter 270:128–142

[CR114] IPCC (2021): The Physical Science Basis. Contribution of Working Group I to the Sixth Assessment Report of the Intergovernmental Panel on Climate Change [Masson-Delmotte, V., P. Zhai, A. Pirani, S. L. Connors, C. Péan, S. Berger, N. Caud, Y. Chen, L. Goldfarb, M. I. Gomis, M. Huang, K. Leitzell, E. Lonnoy, J.B.R. Matthews, T. K. Maycock, T. Waterfield, O. Yelekçi, R. Yu and B. Zhou (eds.)]. Cambridge University Press.

[CR115] von Schuckmann K, Palmer MD, Trenberth KE, Cazenave A et al (2016) Earth’s energy imbalance: an imperative for monitoring. Nat Clim Change 6:138–144. 10.1038/nclimate2876

[CR116] Vondrák J, Burša M (1977) The rotation of the earth between 1955.5 and 1976.5. Stud Geophys Geod 21:107–117. 10.1007/BF01634821

[CR117] Wallace JM, Battisti DS, Thompson DWJ, Hartmann DL (2023) The atmosphere general circulation. Cambridge University Press, Cambridge, p 406

[CR118] Watkins M, Fu Y, Gross R (2018) Earth’s Subdecadal Angular Momentum Balance from Deformation and Rotation Data. Sci Rep 8:13761. 10.1038/s41598-018-32043-82017 30213997 10.1038/s41598-018-32043-8 PMC6137097

[CR119] Whaler KA, Hammer MD, Finlay CC, Olsen N (2022) Core surface flow changes associated with the 2017 Pacific geomagnetic jerk. Geophys Res Lett 49(15):e2022GL098616. 10.1029/2022GL098616 10.1029/2022GL098616 PMC9539959 36247515

[CR120] Yang Y, Song X (2023) Multidecadal variation of the Earth’s inner-core rotation. Nat Geosci 16:182–187. 10.1038/s41561-022-01112-z

[CR121] Yu N et al (2021) Analysis of relationships between ENSO events and atmospheric angular momentum variations. Earth Space Phys 8:e2021EA002030. 10.1029/2021EA002030

[CR122] Zotov L, Bizouard C, Shum CK (2016) A possible interrelation between Earth rotation and climatic variability at decadal time-scale. Geod Geodyn 7(3):216–222. 10.1016/j.geog.2016.05.005

[CR123] Zotov L, Bizouard C, Sidorenkov N, Ustinov A, Ershova T (2020) Multidecadal and 6-year variations of LOD, FAPM. J Phys 1705:012002. 10.1088/1742-6596/1705/1/012002

